# Neutral evolution of Protein-protein interactions: a computational study using simple models

**DOI:** 10.1186/1472-6807-7-79

**Published:** 2007-11-19

**Authors:** Josselin Noirel, Thomas Simonson

**Affiliations:** 1 Laboratoire de Biochimie, École polytechnique, route de Saclay, 91128 Palaiseau Cedex, France

## Abstract

**Background:**

Protein-protein interactions are central to cellular organization, and must have appeared at an early stage of evolution. To understand better their role, we consider a simple model of protein evolution and determine the effect of an explicit selection for Protein-protein interactions.

**Results:**

In the model, viable sequences all have the same fitness, following the neutral evolution theory. A very simple, two-dimensional lattice representation of the protein structures is used, and the model only considers two kinds of amino acids: hydrophobic and polar. With these approximations, exact calculations are performed. The results do not depend too strongly on these assumptions, since a model using a 3D, off-lattice representation of the proteins gives results in qualitative agreement with the 2D one. With both models, the evolutionary dynamics lead to a steady state population that is enriched in sequences that dimerize with a high affinity, well beyond the minimal level needed to survive. Correspondingly, sequences close to the viability threshold are less abundant in the steady state, being subject to a larger proportion of lethal mutations. The set of viable sequences has a "funnel" shape, consistent with earlier studies: sequences that are highly populated in the steady state are "close" to each other (with proximity being measured by the number of amino acids that differ).

**Conclusion:**

This bias in the the steady state sequences should lead to an increased resistance of the population to environmental change and an increased ability to evolve.

## Background

Modern genomics and molecular biology have transformed our understanding of molecular evolution. The diversity of modern proteins is illustrated by the millions of known gene sequences and thousands of known protein structures. It has become clear that proteins are remarkably robust with respect to mutations, retaining structure and function in many cases. This has helped renew interest in theories of evolution that explore the role of "neutral" mutations. A mutation is selectively neutral if it leads to an organism that is viable, but does not increase or decrease the fitness [1,2]. A mutation in non-coding DNA will often be neutral. A mutation in a protein coding sequence can also be neutral if it does not significantly affect the structure, stability, or biochemical function of the protein.

In recent years, computer models have proved useful to explore some of the principles of molecular evolution. To model evolution at the molecular level requires that molecular sequences and structures be explored together. This suggests that simple models should be used, so that a precise mapping can be defined between genotype, phenotype, and fitness [3]. An important example is the use of lattice models to represent protein structures. In these models, the polypeptide is treated as a chain of beads, with one bead per amino acid. The allowed conformations are defined by a simple two- or three-dimensional lattice, with the beads occupying nodes of the lattice. Lattice models first revealed, for example, that for a typical small protein, only a few sequences fold rapidly into a well-defined, stable structure. These sequences tend to adopt structures that have a high stability and are especially "robust" with respect to point mutations: many amino acids can be mutated without disrupting the folded structure. The corresponding folded conformation is said to be highly "designable", since it is shared by many different sequences (corresponding to all the allowed point mutations) [4-6].

In recent years, both on-lattice and off-lattice models of protein structure have been employed in evolutionary models [7-10]. The simplest models focus on a particular protein, and allow evolution only through random point mutations. Such models usually define a set of viable sequences, immersed in a "sea" of non-viable sequences. An individual that undergoes a mutation to a non-viable sequence dies. The set of viable sequences can be thought of as a cluster within the larger space of all possible sequences (for the protein of interest). The set of viable sequences is often viewed as a graph, or network, where the sequences are the nodes, and a point mutation between two viable sequences defines an edge connecting the sequences. In an evolving population, the population dynamics can be viewed as a set of individuals randomly diffusing over the graph [1,11,12].

In a neutral evolutionary model, all the viable sequences have the same fitness. Therefore, the graph just defined is referred to as a "neutral network". We noted above that some viable sequences are especially "robust" with respect to point mutations. In fact, with a neutral evolutionary model limited to point mutations, the steady state has a remarkable property: sequences with a high tolerance of mutations are overrepresented within the population, compared to a random selection of viable sequences. There is a corresponding depletion in sequences that have a low tolerance of mutations, since they undergo a larger proportion of lethal mutations. Furthermore, within the set of viable sequences, the mutationally robust sequences are "close" to other robust sequences: a small number of mutations is needed to transform one into the other. Consequently, the most robust sequences form one or more clusters within the neutral network [7,13]. These clusters are referred to as funnels (or "superfunnels" [7,13]), because they act as a basin of attraction for the population dynamics: in the steady state, population accumulates in these basins. In contrast, sequences that are not very robust to mutations lie mostly outside these regions, forming the outer "edge" of the neutral network. Sequence funneling was recently observed experimentally by directed evolution [14].

Because protein functionality is very complex, evolutionary models usually assume that protein structure can be used as a proxy for function: proteins that adopt the correct structure are assumed viable [10,15]. More recently, explicit models of functionality have been introduced, involving the ability of the protein to bind a small ligand [16-19].

While these models have been very useful, it is increasingly clear that most proteins must interact with other proteins to function, and co-evolve with them [20,21]. The set of Protein-protein interactions has been studied exhaustively for several organisms, and some of its topological properties established [22,23]. Its complexity is thought to correlate with the overall complexity of an organism.

Here, we extend previous evolutionary models to take into account explicitly the essential role of Protein-protein interactions. We model the neutral evolution of two proteins, coupled by a selection criterion that requires the formation of a specific Protein-protein interaction (with a specific, predefined interaction mode). We require only a transient interaction, present around 10–20% of the time. This is meant to mimic the behavior of proteins involved in information transfer and signalling, rather than proteins involved in long-lived, multi-protein complexes. We only consider neutral evolution through point mutations. This mechanism, though simple, is nevertheless important for the evolution of individual protein domains. More complex events like recombination, essential for the creation or rearrangement of entire domains in higher organisms, are neglected here. Protein structure is represented through two simple, very different models: a two-dimensional lattice model and a three-dimensional off-lattice model. The structural models are thus highly simplified and are sometimes referred to as "toy" models. Nevertheless, these and similar models have been shown in the past to provide useful insights. The main qualitative results below appear to be robust with respect to model details. In particular, we have done detailed studies of "monomeric" evolution with several different amino acid alphabets and interaction models that will be published elsewhere.

Only limited studies of Protein-protein pairs have been reported [24]. Here, a functional coupling between two proteins is considered. The two proteins must not only fold, but specifically associate to perform a vital function. Thus, both the stability of the individual monomers and that of the dimer are subjected to negative selection. In contrast to most earlier protein-ligand studies, the two proteins are both allowed to evolve. Using the 2D lattice description of the structures, the chemical equations for dimerization can be solved exactly for any particular sequence pair. Pairs that have a sufficient dimerization ability are viable. The viable sequences can thus be enumerated and the evolutionary dynamics characterized. Viable sequences, which ensure folding of the two partners along with a sufficient degree of dimerization, are all assumed to be equally fit. We refer to this as a neutral evolution model, in the spirit of several models studied by Kimura [1,12]. Under conditions of moderate selection, where only weak dimerization is required, we find that neutral evolution increases the functional effectiveness of the proteins considered: the steady state population is enriched in sequences coding for proteins that readily dimerize. Using a more realistic, 3D, off-lattice description, a similar effect is observed. This result is analogous to the result described above for individual proteins: the (monomeric) steady state was enriched in mutationally robust sequences. In both the monomeric and dimeric cases, sequences in the core of the neutral network are overpopulated, while sequences at the edge are rare. Depletion of sequences at the edge leads to a reduced mutational load [1]. In practice, it has the same effect as a positive adaptation: an enhanced functional ability. The enhancement emerges from a neutral model that requires only a minimal ability to function, through the funneled shape of the network of viable sequences.

## Results

### Sequence diversity and the pressure to dimerize

Two structural models of a protein were considered in this work: a 2D and a 3D model. With either model, acceptable sequence pairs are those that not only fold, but also form a functional dimer with a sufficient cellular concentration (see Methods). In this section, we consider how the selective pressure to dimerize affects the sequence diversity.

### 2D on-lattice proteins

Monomer evolution has been extensively studied with the 2D lattice model [10,25]. Using this model, 12,386,286 out of 33,554,432 sequences fold into one of the 1081 possible conformations (unrelated by symmetry) [8]. The fraction of sequences able to fold (about 37%) is unrealistically high, compared to real proteins. This is due to the simple HP model and the limited space of allowed conformations. Ten conformations are especially robust towards mutations, or "designable", with neutral networks of 40,000–68,000 sequences. We consider that the complexes are formed by associating two square monomers side by side. A pair of such proteins can adopt well over one billion possible sequence pairs (40,000^2^). For these pairs, the selection stringency is characterized by the fractional population *δ *required for the functional dimer *AB*. A value of *δ *= 0.1, for example, means that at chemical equilibrium, the dimer must be present at least 10% of the time. For reasons of computational cost, the analysis is limited to 16 2D dimers. They all involve monomeric neutral networks of about 10,000 sequences. The largest monomeric neutral networks (40,000–68,000 sequences) are too large to allow complete dimerization studies.

**Figure 1:**
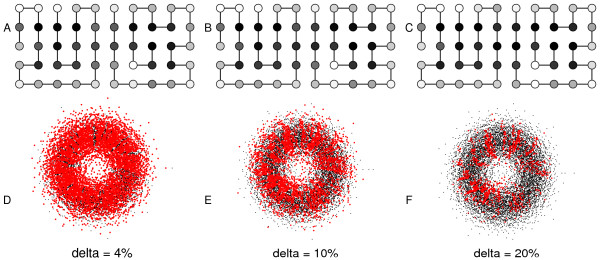
**Example of a 2D dimer**. **A**) Low selective pressure for dimerization: *δ *= 0.04 (i.e., only sequences that lead to a protein fraction of at least 4% engaged in the functional dimer are viable). Amino acids are colored according to the mean sequence in the steady state (hydrophobic: dark; polar: light). **B**) The same dimer under a moderate selective pressure: *δ *= 0.1 This leads to a more hydrophobic interface. **C**) The same dimer with *δ *= 0.2. **D**) The neutral network for one of the protein partners when *δ *= 0.04. Black dots represent viable monomer sequences; red dots represent sequences that survive under the dimerization condition. Connections between sequences are omitted for clarity. The radial position of each sequence reflects its distance from an arbitrary center (the most populated sequence when *δ *= 0). **E**) Idem, *δ *= 0.1. **F**) Idem, *δ *= 0.2.

**Figure 2:**
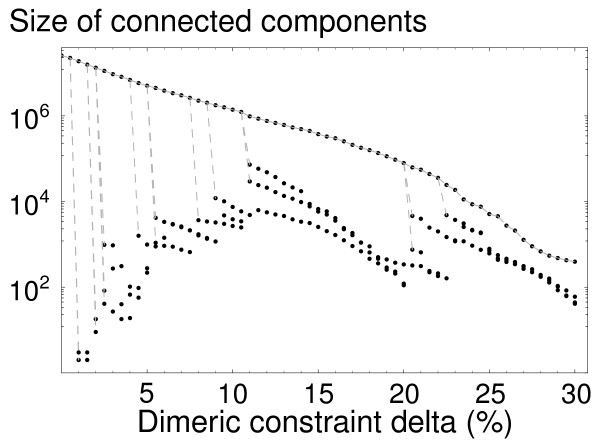
**Size of neutral network components**. For a representative 2D dimer, the size of the four largest components as a function of the selective pressure *δ*. As *δ *increases, there are fewer viable sequence pairs, but there is always a single connected component that is much larger than the other, small components. Dashed vertical lines are visual aids to show how the small components progressively break off from the largest one.

Fig. 1 shows the effect of the selection criterion on a typical dimer. For *δ *= 0, all the pairs of sequences formed from the viable monomeric sequences of A and B are viable. As *δ *increases, sequences that dimerize poorly are increasingly eliminated, and the number of viable sequence pairs decreases rapidly. This decrease is accompanied by a fragmentation of the dimer's neutral network into smaller, disconnected pieces, as shown in Fig. 2. Interestingly, there is always one very large connected component, along with a number of much smaller components. The existence of a single large component implies that many sequences can be explored even though only point mutations are allowed.

The sequences eliminated by selection are those with too few hydrophobic residues at the functional interface. This follows from our energy function (Eq. 3), where hydrophobic-hydrophobic interactions are the most favorable. Indeed, Fig. 1 shows that the average sequence, weighted by the steady state population, has an interface that is increasingly hydrophobic (darker) as *δ *increases (e.g. Fig. 1A,B,C). The neutral network for the pair is increasingly depleted. This is seen by the decreasing number of red dots going from left to right in Fig. 1D,E,F. Despite this depletion, the viable sequences of *A *and *B *remain very diverse: the red dots are not grouped in one part of their respective neutral networks, but are widely distributed throughout the network.

**Figure 3:**
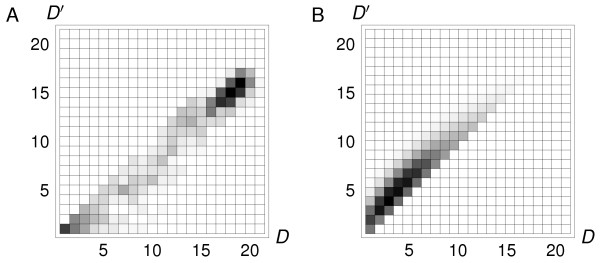
**Diversity of viable genotypes in the neutral networks**. **A**) The neutral network diameters in the absence (*D*) and presence (*D'*) of selection for dimerization, shown as a 2D histogram. We consider each 2D protein structure in turn, with its neutral network of sequences. The dimerization condition (when applied) requires that this protein dimerize specifically with another, particular protein (not shown), chosen arbitrarily. The dimer concentration threshold for viability was set to *δ *= 0.2. The diameter represents the largest "distance" between any two sequences in the neutral network (the number of amino acid mutations that separate them) [23]. The darkest squares are above the diagonal, indicating that among the 1081 structures, most have neutral networks that shrink when the dimerization condition is applied. However the diameters shrink very little: *D' *is typically only 1–2 units (amino acids) smaller than *D*. **B**) The distance between neutral networks in the absence (*D*) and presence (*D'*) of selection for dimerization, shown as a 2D histogram. The dimerization selection criterion is the same as above. The darkest squares are above the diagonal, indicating for most pairs of structures, under the dimerization selection criterion, the corresponding neutral networks shrink so that their mutual distance increases (*D' *> *D*). However, the increase is small, only 1–2 units.

Another, more quantitative measure of sequence diversity is given by the network diameters. The diameter of a neutral network is defined as the largest number of point mutations separating any two viable sequences [23]. In Fig. 3A, the neutral network diameters in the absence (*D*) and presence (*D'*) of selection for dimerization are shown as a histogram. We consider each 2D protein in turn, with its neutral network of sequences (1081 networks in all). The dimerization condition (when applied) requires that these protein dimerize specifically with another, particular protein (not shown), chosen arbitrarily. The dimer concentration threshold for viability was set to *δ *= 0.2. Although the networks shrink when the dimerization condition is applied (many sequences are no longer viable), the diameters shrink very little: *D' *is typically only 1–2 units (amino acids) smaller than *D*. Similarly, the "distance" between two protein folds can be defined as the number of mutations needed to convert one fold into the other. Fig. 3B shows that for the 2D model, the distances between folds increase only slightly (by 1–2 amino acids) under the dimerization condition. In fact, the sequence diversity is such that for moderate values of *δ*, and for typical pairs of 2D proteins, almost every sequence in the neutral network of *A *has at least one *B *sequence with which it can form a viable dimer.

### 3D off-lattice proteins

The second structural model is the three-dimensional, off-lattice model [9,26]. For Grb2 and Vav, it gives 31,469 and 29,667 different HP profiles (according to the classification given in the Methods section). These profiles lead to almost 10^9 ^possible pairs of HP profiles. The selection for dimerization is determined by the *Z*-score of the native, functional structure, compared to the *Z*-score of the decoys. Any pair of sequences whose interaction is weaker than that of the natural sequences is discarded. For the functional structure to be populated at least 10% of the time in the cell, there cannot be more than 9 alternate structures of lower energy. Therefore, the rank *k *we require for the native *Z*-score was varied from 1 to 10. With *k *= 1, only 537 sequence pairs were viable. With *k *= 3–10, there were between 67,291 and 470,334 viable pairs of HP profiles. The latter value represents just one 2000th of all possible pairs. The same reduction is seen in the 2D case with a *δ *of about 0.25 (Fig. 2; Tab. 1). The rather small, viable, 3D fraction is related to the larger size of the 3D dimer interface. The Grb2-Vav complex involves 14–16 amino acids on each partner. A reduction factor of $\frac{1}{2000}$ for the number of sequence profiles can be obtained by fixing the profile (H or P) of just 11 positions in the dimer (since 2^11 ^≈ 2000), or 5–6 positions on each monomer. These positions are chosen according to their proximity to the interface. Fixing 11 positions appears reasonable with respect to the size and diversity of typical Protein-protein interfaces [27].

**Table 1:** Mutational robustness as a function of the functional constraint

*δ*	${\langle n\rangle }_{random}^{A}$	${\langle n\rangle }_{random}^{B}$	⟨*n*⟩_random_	${\langle n\rangle }_{ss}^{A}$	${\langle n\rangle }_{ss}^{B}$	⟨*n*⟩_*ss*_	Survival #	Survival %
0.00	9.18	8.96	18.14	11.48	11.84	23.32	96,108,582	100
0.01	8.47	8.36	16.84	10.95	11.39	22.34	68,290,887	71
0.02	8.03	7.96	16.00	10.61	11.00	21.61	47,695,260	50
0.03	7.67	7.63	15.31	10.30	10.71	21.01	34,273,488	36
0.04	7.43	7.42	14.85	10.10	10.57	20.66	25,785,086	27
0.06	7.06	7.01	14.07	9.69	9.99	19.68	14,825,259	15
0.08	6.71	6.68	13.39	9.38	9.69	19.07	8,983,407	9.3
0.10	6.45	6.42	12.87	9.08	9.40	18.48	5,522,233	5.8
0.15	5.86	5.78	11.64	8.32	8.53	16.86	1,626,428	1.7
0.20	5.18	5.02	10.20	7.48	7.52	15.01	353,538	0.37
0.25	4.43	4.16	8.60	6.21	6.20	12.41	47,192	0.05

Mutational robustness ⟨*n*⟩ as a function of the functional constraint (measured by *δ*). Data are shown for a representative 2D dimer. The two partners each have about ten thousand viable sequences. The mean mutational robustness for randomly chosen sequences is ⟨*n*⟩_random_. The robustness averaged over the steady state is ⟨*n*⟩_*ss*_. These values correspond to the overall mutational robustness of the *AB *dimer. The separate contributions of each partner, *A *and *B*, are also shown. The number and percentage of viable sequence pairs are shown.

Similar to the 2D case, most (70%) of the Grb2 sequences have at least one Vav sequence with which they are able to form a viable dimer. The Vav sequences are less diverse: only 8% of the monomeric sequences survive when *k *= 10. This may be an indication of insufficient sequence sampling during the Monte Carlo simulation of the Vav monomer. Longer (and expensive) simulations are needed to test this further. However, the cost of the present calculations is already close to the limit of what is feasible (weeks of CPU time to construct the dimeric neutral network using ~10 recent processors).

### Independence between mutational robustness and dimerization ability

The selective pressure to expose hydrophobic residues might be expected to correlate with a lower mutational robustness of the two dimerizing proteins. Indeed, the dimerizing sequences are more constrained by negative selection, so that they might have fewer mutations that lead to viable sequences. To quantify this idea, we define more precisely the mutational robustness of a particular viable sequence as the number *n *of its single mutants that are also viable. With respect to the neutral network and its graph structure (see Methods), *n *represents the number of neighboring nodes the node is connected to and can be identified with the "mutational robustness" of the sequence pair. The robustness *n *depends on the protein (A or B), on the particular sequence, and on the level of stringency of the dimerization condition. In the limit where *δ *= 0 (no dimerization required), *n *becomes the mutational robustness of the protein (A or B) considered as a monomer. As *δ *increases, the monomeric networks are increasingly depleted (Fig. 1), and typical values of *n *may be expected to decrease for both A and B. Another useful quantity to characterize a particular sequence is the folding temperature *T*_*f *_of the protein. *T*_*f *_is a measure of protein stability (see Methods), and might also be expected to decrease as the stringency of selection for dimerization increases, since exposing hydrophobic residues tends to lower stability with our energy function.

**Figure 4:**
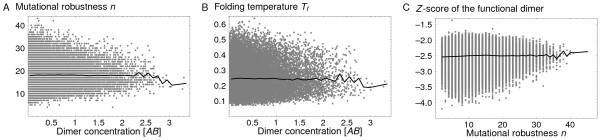
**Absence of correlation of *n *and *T*_*f *_with dimerization ability**. **A**) For a representative 2D protein dimer, we show the mutational robustness *n *of each viable sequence pair, versus the cellular concentration [*AB*]_func _of the functional dimer at chemical equilibrium for that pair. No selective pressure for dimerization is applied. The black line represents the average over the distribution of *n *values for each value of [*AB*]_func_. *n *and [*AB*]_func _are seen to be uncorrelated. **B**) For the same 2D dimer, we show the folding temperature of each viable sequence pair as a function of [*AB*]_func_. **C**) For the Grb2-Vav 3D dimer, we show the *Z *value (which measures the dimerization ability) of each viable sequence pair as a function of *n*. The two are seen to be uncorrelated, even though a selective pressure for dimerization is present in this case (see text).

We consider first the neutral networks of *A *and *B *separately, in the absence of any dimerization requirement, viewing them as two independent monomers. The mutational robustness *n *and the folding temperature *T*_*f *_are defined for each sequence of *A *or *B *as described above. Next, we take an *A *sequence and a *B *sequence; we identify their functional interface (see Methods), and we compute the concentration [*AB*]_func _of the functional dimer in the cell at chemical equilibrium (Eqs. 4). No negative selection is applied for dimerization; i.e., *δ *= 0. Considering all pairs of *A*, *B *sequences, we find that the ability to dimerize is actually not correlated with either *n *(Fig. 4A) or the folding temperature *T*_*f *_(Fig. 4B). Sequences with very diverse values of *n *and *T*_*f *_have the same ability to dimerize, as measured by [*AB*]_func_.

The data in Fig. 4A,B correspond to one representative dimer, made of a particular pair of protein structures. For the 16 dimer structures we analyzed, the correlation coefficients range from -0.026 to 0.042 for *n *(respectively, -0.035 to 0.100 for *T*_*f*_). Inspecting the sequences in more detail, we find that, in fact, dimerization can be enhanced without increasing the number of exposed hydrophobic residues. Instead, hydrophobic residues can be moved to the interface region from another part of the protein surface. Within the simple 2D model, this operation has very little effect on the protein stability and folding temperature, which explains that *T*_*f *_and [*AB*]_func _are uncorrelated. Since *T*_*f *_and *n *are known to be strongly correlated [13], *n *must also be uncorrelated with [*AB*]_func_.

We consider next the neutral networks of *A *and *B *in the presence of a selective pressure for dimerization. For the same 16 representative dimer structures, we consider a series of selection thresholds *δ*. By imposing a particular *δ*, we effectively discard all the points in Fig. 4 to the left of *δ *and all the corresponding sequence pairs. Surviving pairs close to the dimerization threshold tend to lose some of their neighboring sequences, so that their mutational robustness *n *decreases. The net effect is a rather strong correlation between [*AB*]_func _and *n*. For *δ *= 10–30%, the correlation coefficient is about 50–65%. At a low selective threshold of *δ *= 2%, the correlation is about 30%.

For the three-dimensional Grb2-Vav dimer, the correlation between mutational robustness and functionality is very weak, even in the presence of a selective pressure for dimerization. In Fig. 4, the mutational robustness *n *is plotted against the dimerization energy *Z*-score, denoted *Z*. The data correspond to a dimerization selection threshold of *k *= 10, for a total of 470,334 viable dimer sequence profiles. The correlation coefficient between *n *and *Z *is low, less than 5%.

This independence between the *Z *and *n *(or *T*_*f*_) is likely to hold qualitatively for real proteins. For a given dimer interface AB, we expect that the interface sequences will be rather contrained by natural selection [27,28], whereas a wider range of amino acid types may be found on the remaining parts of the surfaces and in the proteins' core, leading to a wide range of protein stabilities. However, a systematic analysis of both sequence conservation and protein stability in families of dimerizing proteins would be needed to make this statement quantitative. Thermodynamic data are scarce, and such an analysis is beyond the scope of this study.

### The steady state is enriched in functional sequences

**Figure 5:**
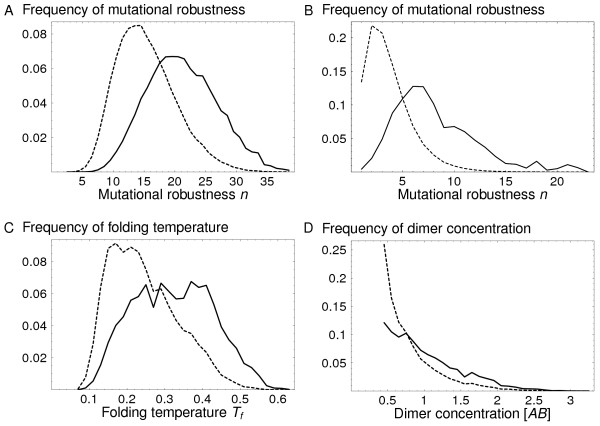
**The population dynamics enhance robustness and functionality**. **A**) The distribution of mutational robustness *n *for a given 2D protein dimer. Solid line: the steady state population. Dashed line: a population drawn randomly from the neutral network. **B**) Idem for the 3D, off-lattice Grb2-Vav 3D dimer. **C**) The folding temperature distribution for the same 2D dimer; solid line: steady state population; dashed line: random population. **D**) The distribution of the equilibrium concentration [*AB*]_func _of the functional dimer, for the same 2D dimer; solid line: steady state population; dashed line: random population.

Previous studies of single protein evolution have revealed an enrichment in mutational robustness in the steady state [7,8,13]. Sequences in the core of the neutral network are overpopulated, while those at the edge of the network, with fewer graph connections, are underpopulated. This steady state enrichment is preserved under the dimerization constraint, as shown in Fig. 5A. The extent of enrichment is similar to the pure monomeric case; see Table 1 for illustrative, numerical values for a particular complex. A similar enrichment is observed for the 3D proteins (Fig. 5B). The agreement between the 2D and 3D models provides encouraging evidence that this behavior does not depend on model details. The dimer folding temperature is also enriched in the steady state (Fig. 5C). This is consistent with the known correlation between *n *and *T*_*f*_.

In a similar way, the cellular concentration of the functional dimer is enriched in the steady state (Fig. 5D). In other words, the sequences that form high affinity complexes are overpopulated. Thus, neutral evolution leads not only to increased mutational robustness, but to increased concentrations of the functional species present in the average cell. This effect and its extent were somewhat harder to anticipate, despite the analogy to the monomeric result (enrichment in *n*). Like the mutational robustness *n *in the monomeric case, [*AB*]_func _is directly selected for in the dimer case. However, the mode of selection is quite different for the two quantities: [*AB*]_func _is subjected to a threshold, while selection for *n *acts in a more continuous manner. In addition, we saw that the concentration of the functional dimer is not correlated with, or closely-related to either *n *or *T*_*f*_. The enrichment in *n *arises because highly-connected sequences are grouped in the middle of the neutral network. In effect, the enrichment arises because *n *varies in a smooth, continuous manner over the network, so that robust sequences are close to other robust sequences. But we saw above that the sequences satisfying the dimerization threshold are widely distributed throughout the underlying monomeric network (Fig. 1). Therefore, it was not obvious ahead of time that dimerization ability would vary sufficiently smoothly and continuously.

**Figure 6:**
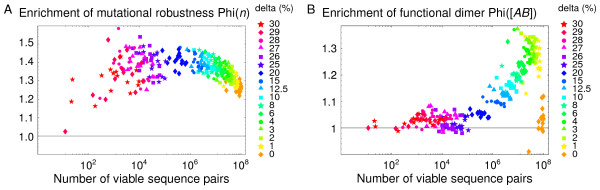
**The population dynamics enhance robustness and functionality**. **A**) Φ(*n*) = ⟨*n*⟩_*ss*_/⟨*n*⟩_random _measures the enrichment in mutational robustness due to the steady state dynamics. Each colored point corresponds to a 2D protein pair subjected to a dimerization condition. Each color corresponds to a particular level of the selection stringency, indicated by the value of the concentration threshold *δ *(in %, legend on right). Data are shown for a selection of 15 dimers. **B**) Φ([*AB*]) = ⟨[*AB*]⟩_*ss*_/⟨[*AB*]⟩_random _measures the enrichment in dimerization ability due to the steady state dynamics: ⟨[*AB*]⟩_*ss *_is the cellular concentration of the functional dimer averaged over the steady state sequences; ⟨[*AB*]⟩_random _is the value averaged over the viable sequences, regardless of their population. A value of Φ([*AB*]) greater than 1 indicates an enrichment of the steady state population in sequences that readily dimerize. Colors indicate the selection stringency. Data are shown for a selection of 10 dimers.

In Fig. 6, we define an enrichment factor for dimerization ability, Φ([*AB*]) = ⟨[*AB*]⟩_*ss*_/⟨[*AB*]⟩_random_, where ⟨[AB]⟩_*ss *_is the cellular concentration of the functional dimer averaged over the steady state sequences, and ⟨[*AB*]⟩_random _is the concentration averaged over all the viable sequences, regardless of their population.

Typical values of Φ([*AB*]) are greater than 1, corresponding to enrichment. The enrichment in functional species is strongest when the selection criterion is only moderately stringent: Φ([*AB*]) ≈ 1.2–1.3 when *δ *≈ 0.01–0.10. As *δ *increases, selection becomes more stringent and the set of viable sequences is increasingly depleted (Fig. 5). Enrichment then decreases. A model with larger proteins and/or a more complex amino acid alphabet would probably allow a greater enrichment, extending to higher selection stringencies. The requirement of a 10% population for the functional dimer (*δ *= 0.10) appears reasonable if the dimer's function is to form transiently and transmit a signal.

For one particular dimer, we constrained protein B to have a single, fixed sequence, so that it no longer evolves and plays the role of a simple ligand. In this case, the steady state enrichment in the concentration of the functional dimer is reduced by half (Φ([*AB*]) ≈ 1.15 instead of 1.3; data not shown).

For the 3D model, it is harder to characterize the enrichment (if any) in steady state dimerization ability, because there are too many (784) possible structures and the dimer concentrations cannot be readily computed. Nevertheless, the steady state populations of the viable sequences are available, so that we can compare the typical *Z*-scores, *Z*, in the steady state population and a random population. This is done in Table 2. An enrichment factor Φ(*Z*) is defined in the same way as Φ([*AB*]), above. We considered selection thresholds *k *between 2 and 10. We recall that a value of *k *= 4 implies that the functional dimer is among the four lowest-energy structures, out of a total of 784 structures. As *k *increases, Φ(*Z*) first increases from 1.07 to 1.18 (*k *= 4 or 5), then decreases to 1.04 (*k *= 9 or 10). As in the 2D case, the enrichment is maximal for an intermediate selection stringency. The maximum enrichment factor is roughly comparable in the 2D and 3D cases, even though the measures of dimerization ([*AB*]_func _and Z) are obviously different. Again, the qualitative 2D-3D agreement is encouraging.

**Table 2:** Stability enrichment for the Grb2-Vav 3D dimer

*k*	viable sequences	⟨*n*⟩_*ss*_	Φ(*Z*)
1	537	14.13	1.05
2	30801	4.65	1.07
3	67291	3.50	1.08
4	109954	3.07	1.18
5	157903	3.07	1.18
6	211133	3.01	1.06
7	269022	3.00	1.07
8	331852	2.96	1.07
9	398776	2.99	1.04
10	470334	3.00	1.04

Steady state enrichment factor Φ(*Z*) of the dimerization ability (measured by the *Z*-score) for the Grb2-Vav 3D dimer as a function of the functional constraint (measured by *k*). The number of viable sequence profile pairs and the mean mutational robustness ⟨*n*⟩_*ss *_are also shown.

### Functional sequences form a funnel

**Figure 7:**
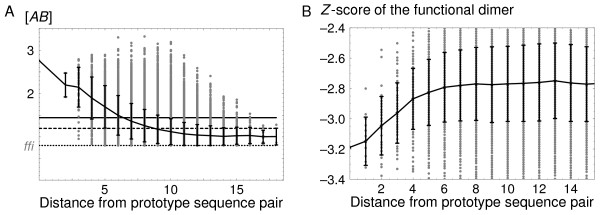
**Emergence of a "functional funnel" in sequence space**. **A**) 2D dimer: The mean concentration [*AB*]_func _of the functional dimer as a function of the distance of each viable sequence pair from the prototype pair (the most populated pair in the steady state). Data are shown for a representative dimer and a selection threshold of *δ *= 0.08 (dotted horizontal line). There are no sequence pairs below the dotted line, because such sequences are not viable, by definition. Black curve: the mean value for each distance. Black vertical bars indicate the standard deviation at each distance. Dashed horizontal line: average over a random set of sequences. Solid horizontal line: overall steady state average. The concentration [*AB*]_func _varies widely, but the mean value drops off rapidly and smoothly as one moves away from the prototype sequence pair. **B**) Similar representation for the 3D Grb2-Vav dimer: the dimerization *Z *score as a function of the distance from the prototype sequence.

The enrichment in functional species is strongest for sequence pairs near the "prototype" pair, defined as the most populated pair in the steady state [8]. This is illustrated in Fig. 7A for the 2D proteins. The mean concentration [*AB*]_func _of the functional dimer is plotted for each viable sequence pair, as a function of its distance from the prototype pair (for a representative dimer and a few values of the selection threshold, *δ*). The concentration [*AB*]_func _varies widely, but the mean value drops off rapidly and smoothly as one moves away from the prototype sequence. Similar behavior is seen for other 2D dimers. Thus, the sequences responsible for the functional enrichment are grouped in the center of the neutral network, forming a basin, or funnel in sequence space.

With the 3D model, dimerizing ability is measured by the *Z*-score. We saw above that the corresponding enrichment factor, Φ(*Z*), was slightly smaller than Φ([*AB*]) in the 2D case. Nevertheless, a basin of high affinity complexes is also seen with the 3D model, although the funnel shape is somewhat less pronounced (Fig. 7B). The funnel shape flattens out at a distance of about 6–7 from the prototype sequence. A small number of sequences forming high affinity complexes are actually found outside the basin, at distances of 13–14 from the prototype.

## Discussion

Protein-protein interactions are central to cellular organization, and must have appeared at a very early stage of evolution. To understand better their effects, we considered here two simple, "toy" models of protein structure and evolution, and determined the effect of explicitly selecting for Protein-protein interactions. By employing a 2D, lattice representation of protein structure and binary, hydrophobic/polar sequences, exact calculations could be performed. The 3D, off-lattice model gives a similar qualitative picture. For example, the 3D model also predicts that mutationally robust sequences are overrepresented in the steady state, in agreement with the well-known result of lattice models [7,13,29].

Lattice models like the present one have been a subject of debate, because of their use of limited alphabets [25,30], the restriction to maximally compact structures [31,32], and the use of a highly-simplified energy function [33]. Despite their simplicity, these models have some important protein-like features, such as hydrophobic-polar segregation. The simple, pairwise energy function allowed England and collaborators to devise a determinant of protein designability that is applicable to real proteins [5]. Nonetheless, artifacts certainly arise from these models, and that is why we also used a significantly different, 3D, off-lattice structural model, to corroborate the conclusions drawn from the lattice model.

Here, a functional coupling between pairs of genes was added to two previous evolutionary models: the two proteins of interest must associate in order to function. The steady state enrichment in mutational robustness is preserved under this additional constraint. Sequence diversity remains very large when dimerization is required, even though only a fraction of sequences survive under these more selective conditions. The sequence diversity is reflected, for example, by the wide range of protein robustnesses and folding temperatures that can lead to the same dimerization ability. It is somewhat unexpected that as the pressure to dimerize is increased and more and more sequence pairs are eliminated, the viable sequences continue to be largely grouped in a single, continuous network (Fig. 2), instead of splitting into many small, disconnected networks. If one of the proteins were constrained to have a fixed ligand, so that it no longer evolves but functions as a simple ligand, this property would probably not hold. A single, continuous network makes it easier to explore sequence space, since single mutations can be used more extensively, rather than large hops involving several mutations at a time.

The present treatment corresponds to neutral evolution, in the sense that it treats all viable sequences as equally fit. The model has a at fitness plateau–the neutral network, surrounded by a sea of non-viable sequence pairs. Evolution takes the form of a random diffusion throughout the neutral network. This neutral picture should be in rough qualitative agreement with real proteins. Neutral mutations are very common in proteins, as shown by the sequence diversity associated with modern protein folds. The neutral model predicts that the probability for a protein to retain its native fold decreases exponentially with the number of mutations, at least for the first few mutations; this prediction agrees with recent experimental observations [34]. The proportions of tolerated mutations computed here for the individual "proteins" are also comparable to those of several real protein folds [35].

Our model selects for a minimal level of functional ability, determined by the chosen dimer concentration threshold, *δ*, or the *Z*-score rank, *k*. The steady state dynamics then lead to a population that is enriched in sequences that form high affinity dimers, well beyond the minimal ability needed to survive. In other words, the functionality of the typical sequence pairs has been enhanced by the evolutionary dynamics. The enhancement occurs through negative selection, and can be viewed as a reduction of the mutational load [1]. In practice, it has a similar effect to the adaptation that would occur in response to positive selection; namely, enhanced functionality. The functional enrichment arises because of the plateau form of the neutral network and because of the funneled organization of the sequences within the network. The enrichment is analogous to the enrichment in mutational tolerance seen previously for single protein models. Nevertheless, the extent of the enrichment and its qualitative response as a function of the stringency of the selection needed to be investigated. Indeed, the mode of selection for mutational robustness and that for dimerization are mathematically quite different. Dimer sequences are widely dispersed throughout the monomeric networks, whereas the steady state enrichment in a given property (mutational tolerance or dimerization ability) is related to its continuity over the set of viable sequences. Little or no correlation is seen between the "monomeric" properties, *n *and *T*_*f*_, and the dimerization ability. In addition, while the functional enrichment first increases with *δ*, it then decreases for larger values of *δ*. Back-of-the-envelope predictions for dimers are difficult because of the complex chemical equilibria involved (see Methods).

The timescale of the present model is set by the mutational probability per unit time, *α *in Eq. 2. The enrichment in [*AB*]_func _is then obtained in the evolutionary steady state. In real systems, the timescale to reach the steady state depends on the population size *N *and the mutation rate, *μ*. Previous simulations have identified two regimes, characterized by the product *N μ *[36]. When *N μ *> 100 (large population and/or mutation rate), the population is expected to rapidly sample the steady state, so that the enrichment phenomena predicted above should be visible. For lower populations and/or mutation rates, *N μ *≪ 100, the population is expected to behave like a random sample drawn from the neutral network, so that no enrichment should be observed. If *μ *is a mutation rate per individual, then *N μ *represents the mutation rate within the entire population. The product *N μ *is small for eukaryotic populations and large for RNA viruses. For eubacteria with a generation time of minutes, a neutral mutation should appear in a typical protein about *N μ *= 10^1^–10^3 ^times a day within a large colony [37]. The given range corresponds to different colony sizes (10^5^–10^7 ^individuals); it can be expanded if one considers longer generation times or artificially accelerated mutation rates (e.g., in the presence of chemical mutagens). This range for *N μ *should encompass the two regimes just discussed [36]. Thus, the role of the steady state dynamics in elevating the average functionality could be experimentally tested by comparing two such bacterial colonies. Some of our predictions could also be tested by analyzing experimental protein sequences. The weak correlation between dimerizing ability and protein stability is in accord with our knowledge of Protein-protein interfaces. Typical Protein-protein interfaces have a few amino acids forming a central hydrophobic patch; small, polar, mutational "hotspots" are also frequent [27]. The evolutionary contraints on these local surface patches should have a limited effect on other surface and core regions, so that a large range of protein stabilities can be achieved despite the constraints. Conversely, it would be interesting to compare the dimerization abilities of very stable proteins, such as those of thermophilic organisms.

## Conclusion

From the present models, the sequences that are populated in the steady state are enhanced in their functional ability. This should allow an increased resistance to environmental change, or adaptability [14]. Indeed, a strong dimer is more likely to be preserved under a change in the surrounding temperature or pH, for example. They should also provide an increased ability to evolve and comply with newly imposed functional requirements. Indeed, after a gene duplication event, a protein *A *that starts out with the ability to bind strongly to its partner *B *will be better able to explore mutations that allow it to co-evolve with *B*, or to dimerize with other, existing, homologues of *B *(using the ancestral binding mode, at least at the beginning). This effect, which arises from a very simple, minimal model of protein evolution, should lead to an enhanced ability to create homologous interacting pairs of proteins, and could have played a role in the early emergence of Protein-protein interaction networks.

## Methods

### The evolutionary model and its properties

Following [3,8] and others, we first assume that all genes evolve independently, and we focus arbitrarily on one of them. In a second step, below, we will consider co-evolution of two interacting proteins. For now, the single gene of interest is assumed viable if the corresponding protein folds into its correct conformation. The *S *sequences that adopt this conformation are all assumed to be equally fit. We assume evolution can only occur through point mutations; i.e., substitutions of a single amino acid. Frameshift and nonsense mutations are assumed to be lethal. The complete set of viable sequences defines a graph, containing *S *nodes. Each node represents a viable sequence; links between nodes represent point mutations. The graph may not be fully connected; i.e., it may be impossible to connect two viable sequences by a series of point mutations. If the entire population starts out with the same, "native" protein sequence, then future evolution will only explore the corresponding, connected subgraph. Therefore, we can assume without loss of generality that there is only one connected graph, which is referred to as a "neutral network".

The following, discrete-time, evolutionary model is analyzed [3,8]. For simplicity, we describe it in detail for the present, single gene case. The case of interacting proteins is considered further on. The model behavior is simplest in the case of a very large (essentially infinite) population, and we limit ourselves to this case. The effect of a finite population size is considered in the Discussion. Between times *t *and *t *+ 1, an individual with sequence *i *has a probability *α *of undergoing a point mutation and a probability *β *of dying. A new individual has a probability *γp*_*i *_of appearing spontaneously by birth; with probability 1 - (*α *+ *β *+ *γ*), the individual continues unchanged. After each generation, populations are rescaled to maintain a constant total. The probability to find a given individual with sequence *i *at time *t *is denoted *p*_*i*_; the change between *t *and *t *+ 1 is denoted *δp*_*i*_. We consider here the limit of a large population, in which case these probabilities follow the equation:

$$\delta p_{i}=-(\alpha +\beta -\gamma )p_{i}+\frac{\alpha }{M}\sum_{k\sim i}p_{k}.$$

Here *k *~ *i *means that *k *and *i *are neighbors in the neutral network and *M *= 25 denotes the chain length of the protein. The *S *× *S *adjacency matrix *C *[23] is defined by: *C*_*ij *_= 1 if *i *~ *j *and zero otherwise; the vector of sequence probabilities is *p *= (*p*_1_, *p*_2_, ... , *p*_*S*_). Eq. 1 can be rearranged into the following vector form:

$$\delta p=\frac{\alpha }{M}(C-\nu I)p.$$

It is easy to show that *ν *is the mean number of neighbors of the sequences in the network: $\nu =\sum_{i=1}^{S}\nu_{i}p_{i}$, where *ν*_*i *_is the number of neighbors of sequence *i*. Eq. 2 describes the flow of population within the neutral network. The first term in parentheses on the right represents sequences flowing into a given node *i*; the second term represents sequences owing out of *i*, taking into account the fraction of viable and lethal mutations. An important property of Eq. 2 is that there is a single stable steady state. The steady state probability vector, *p *= *p*^*ss*^, is an eigenvector of *C*, associated with the largest eigenvalue, *ν *= *ν*^*ss*^. Remarkably, the steady state can be shown to be not only stable with respect to small fluctuations, but globally stable. A detailed proof will be published elsewhere; see [3] for a detailed treatment of related mathematical models.

### Structural models: 2D lattice model and 3D off-lattice model

Two physical models of a protein are considered. The first treats the "protein" as a chain of *L *= 25 beads, or amino acids, which can be either polar (P) or hydrophobic (H). Acceptable conformations occupy a two-dimensional, 5 × 5, square lattice. Thus, only maximally compact conformations are allowed. The energy is

$$E=\sum_{i<j}e_{ij}\Delta_{ij},$$

where Δ_*ij *_= 1 if the beads *i*, *j *are neighbors on the lattice and zero otherwise, and the interaction coefficients depend on the type (P or H) of each bead. The values *e*_HH _= -2.3, *e*_HP _= -1, and *e*_PP _= 0 are used, following [8,38], to favor compact conformations with a hydrophobic core. A particular sequence is considered to fold if its lowest energy conformation is unique (i.e., non-degenerate). It is viable if it folds into a particular, preselected conformation. The protein chain is considered to have a direction (even though the energy function does not); e.g., the sequences HPP and PPH are different. Sequence exploration is done by exhaustive enumeration.

With the 2D model, we can calculate exactly the folding temperature *T*_*f *_of each structure and sequence. By definition, *T*_*f *_is the temperature at which the native conformation is populated 50% of the time. It is straightforward to compute it numerically from Boltzmann's law and the energy spectrum of the 1081 possible conformations. Because we consider only the maximally compact conformations during the computation, the value of *T*_*f *_is overestimated. For a protein dimer, we define *T*_*f *_as the minimum of the folding temperatures of the two separate partners.

The second physical model is a three-dimensional, off-lattice model [9,26]. Two proteins are considered: the 69-residue SH3 domain of Vav and the 57-residue SH3 domain of Grb2. These two form a Protein-protein complex (PDB accession number 1gcq). For each one, the experimental, 3D structure is considered, along with over 1200 "decoy" structures, whose backbone geometries are taken from completely different proteins [39,40]. The sidechains are built assuming the most common rotamer for each amino acid type [41]. For each protein, 100 additional decoys, with more native-like structures, were produced by molecular dynamics *in vacuo *at 310 K. Amino acids interact through Eq. 3, with Δ_*ij *_= 1 if they have two nonhydrogen atoms within 4.5*Å *of each other, and zero otherwise. The amino acids are divided into two classes: H = {LVIMCASTPGFWY} and P = {EDNQKRH}. The first, "hydrophobic" class includes amino acids usually considered hydrophobic or neutral; the second class includes amino acids considered polar.

The energy parameters are *e*_HH _= -8.5, *e*_HP _= 9.0, *e*_PP _= -3.5, These parameters were optimized to discriminate experimental protein structures from large sets of decoys [39,40]. With this model, we first enumerate sequences that are viable as monomers; i.e., they fold into the desired, native structure. For this step, the monomer sequence space is explored by a Monte Carlo method [9]. A "move" consists in a random point mutation, which is accepted if the desired, functional structure has a sufficiently low energy, compared to the non-functional, decoy structures. Specifically, the functional fold must have an energy gap (energy difference from the lowest decoy) and a Z-score (energy difference from the average decoy, in standard deviation units) as large as those of the starting sequence. The starting sequence is slightly different from the native sequence. It is obtained by minimizing the latter through several thousand Monte Carlo moves. A trajectory of one hundred million mutations is then performed. For each accepted mutation, we also explore systematically its nearest "neighbors" (all its single mutations), thus generating a large, representative subset of the relevant neutral network in monomeric sequence space [9].

Once the neutral network has been constructed (either for a 2D or a 3D protein), the steady state distribution of sequences is computed by an iterative, shifted power method [42], which yields the eigenvector of *C *corresponding to the largest eigenvalue.

### Interacting genes: the 2D on-lattice case

In a second step, an evolutionary scenario with interacting genes is explored. We describe first the 2D lattice case. It is assumed that a vital function can only be performed when two proteins *A*, *B *not only fold, but specifically dimerize. The sequences that are viable as monomers are first obtained by the procedure described above. In addition, the two proteins, because of their square-lattice structure, can form ten homodimers *AA*, *BB *and 16 heterodimers *AB*, just one of which is functional. Inter-protein interactions are described by Eq. 3. In addition, dimerization is opposed by a constant entropic penalty, *ε*. We consider here only sequences that are known to form viable monomers (see above), so that their unfolded conformations are unstable and can be neglected. We only consider association between the two proteins *A *and *B*; association of *A *with a third protein *C*, corresponding to a different 2D structure, is largely neglected. We can always view the pool of other proteins *C*, *D*, ... as the source of a competing, background interaction. Our model can incorporate these interactions only in an average way, by replacing the protein pool by a single competing protein, whose sequence is an average over all viable monomer sequences of all structures *C*, *D*, ... In that case, the competing proteins have the same effect as a modification of the total concentration of proteins *A *and *B*. In what follows, we always explore a wide range of protein concentrations, and so interactions with other proteins are not considered further. There are then 38 possible chemical species, whose equilibrium concentrations are obtained by solving the system:

$$\begin{array}{ll} {}[AA_{I}]=a_{I}{\left[A\right]}^{2}, & 1\leq I\leq 10\\ {}[BB_{J}]=a_{J}{\left[B\right]}^{2}, & 1\leq J\leq 10\\ {}[AB_{K}]=c_{K}[A][B], & 1\leq K\leq 16 \end{array}$$

with fixed total concentrations [*A*]_tot _and [*B*]_tot_. Here, *a*_*I*_, *b*_*J*_, *c*_*K *_are equilibrium constants; for example *a*_*I *_= exp(-Δ*E*_*I*_/*kT*), where *k *is Boltzmann's constant, *T *the temperature, and Δ*E*_*I *_is the association free energy of the dimer *AA*_*I*_. The *a*_*I*_, *b*_*J*_, *c*_*K *_depend on the sequences of *A*, *B *through the association free energies. The chemical equations 4 can be reduced to a fourth-order polynomial, by grouping all the *AA*_*I *_(respectively, *BB*_*J *_or *AB*_*K*_) dimers into a single species A(resp., ℬ, C) and solving for them. The relative concentrations of the various *AA*_*I *_subspecies, for example, are then obtained immediately from [A]. The whole system can thus be solved numerically very efficiently. An *A*, *B *pair with particular sequences is then considered viable if the functional dimer has an equilibrium concentration greater than a chosen threshold *δ*. The functional dimer is the one that minimizes the dimerization energy, averaged over all the *A*, *B *sequences that fold (into their designated native conformations).

### Interacting genes: the 3D off-lattice case

We now turn to the 3D, off-lattice case. We consider the Grb2-Vav complex [43] which plays a role in tissue specific signaling in the hematopoietic lineage [44]. In contrast to the lattice case above, there are far too many possible dimer structures for an exact enumeration to be done. Instead, we consider a limited set of dimeric decoy structures. These were generated by a docking procedure described in detail elsewhere [40]. Briefly, we start from the two separate proteins, with their native sequences, positioned randomly with respect to each other. They are then docked together with a molecular mechanics energy function [45], using restrained energy minimization. In an initial phase, the restraint consists in a harmonic spring that pulls their centers of mass together. In a second phase, the restraint corresponds to an electrostatic contrast introduced artificially between the two proteins: charges on one are slightly increased; charges on the other are slightly decreased. The pair is energy-minimized, allowing for limited intra-protein deformations. Structures that involve too large a deformation of either partner (rms deviation of more than 3.5*Å *with respect to the starting monomer conformation) are discarded. Overall, we produced a total of 1695 decoys, of which 912 were discarded because they lead to a lower interaction energy than the native structure. This is due to the simplicity of the energy function. We are left with 783 decoys (compared to 35 non-native structures in the 2D dimer case).

To determine the viable dimer sequences, we start from the sequences that are viable as monomers, generated by the Monte Carlo method described above. Because the number of accepted monomer sequences is very high, we only keep one sequence per hydrophobicity profile (which is computed using the classification shown in the Methods section), picked arbitrarily from the available sequences. There were a total of 31,469 and 29,667 profiles for Grb2 and Vav, respectively. We then consider the ability to dimerize, by comparing the energy of the native dimer structure to the energy of all the decoy structures. For a given pair of Vav and Grb2 sequences and a given dimer structure, the energy is obtained by threading the sequence onto the dimer structure. Sidechains are positioned in their most common rotamer, as described above for the monomer case. For a given sequence pair, a *Z*-score is calculated for each dimeric structure. The *Z*-score is defined as the energy of the structure relative to the average energy, measured in standard deviation units. A pair of sequences is considered to form a viable dimer if the native structure has a sufficiently low *Z*-score. Specifically, its *Z*-score should be among the top *k *values, where *k *is an integer between 3 and 10. Choosing *k *= 3, for example, means that for a sequence pair to be viable, at most two decoys should have a lower *Z*-score than the native dimer structure. By varying *k*, we can explore different stringencies for the selection criterion. This is analogous to varying *δ *in the 2D lattice case. With *k *= 10, there are 470,334 viable pairs of HP profiles. To test the viability of the sequences pairs required several weeks of CPU time using ten computer processors. Once the viable sequences are known, the steady state is computed as for the monomer case.

## Authors' contributions

TS conceived of the study and its design, and participated in the numerical implementation. JN participated in the design and did the bulk of the programming, computations, and analysis. JN and TS drafted, wrote, and approved the manuscript.
